# Clinical and laboratory characterization of adult sickle cell anemia patients in Kinshasa

**DOI:** 10.1371/journal.pone.0278478

**Published:** 2022-12-16

**Authors:** Paul Kabuyi Lumbala, Gloire Mbayabo, Mamy Nzita Ngole, Aimé Lumaka, Valerie Race, Gert Matthijs, Chris Van Geet, Prosper Tshilobo Lukusa, Koenraad Devriendt, Tite Minga Mikobi

**Affiliations:** 1 Department of Pediatrics, University of Kinshasa, Kinshasa, DRC; 2 Department of Clinical Biology, University of Kinshasa, Kinshasa, DRC; 3 Faculty of Medicine, Center of Human Genetics, University of Kinshasa, Kinshasa, DRC; 4 Center for Human Genetics, KU Leuven and University Hospitals Leuven, Leuven, Belgium; 5 Department of Cardiovascular Sciences, Center for Molecular and Vascular Biology, KU Leuven, Leuven, Belgium; 6 Faculty of Medicine, Molecular Biology and Human Genetics Department of fundamental sciences, University of Kinshasa, Kinshasa, DRC; University of Illinois at Chicago, UNITED STATES

## Abstract

**Background:**

Sickle cell anemia (SCA) is a monogenic hemoglobinopathy associated with severe acute and chronic complications, with the highest incidence worldwide in Sub-Saharan Africa. The wide variability in clinical manifestations suggest that a uniform response to hydroxurea may not be attained. In view of a potential treatment with hydroxyurea (HU), we assessed the variability of clinical and hematological manifestations in a cohort of adults with SCA in Kinshasa, capital of the DR Congo in Central Africa.

**Methods:**

A cross-sectional study was conducted in a hospital dedicated to SCA management in Kinshasa. Clinical history of patients was recorded, a complete physical examination performed. The diagnosis was confirmed by means of DNA analysis. A full blood count and hemolysis markers were measured. The severity of the disease was evaluated by means of a previously reported score.

**Results:**

The study group consisted of 166 genetically confirmed SCA patients. The SCA severity was mild in 28.9%, moderate in 64.5% and severe in 6.6%. The disease severity score increased with patient’s age (p ≤ 0.001). The severity was higher in males compared to females (p = 0.012). In males, the severity score was correlated with the presence of priapism (p = 0.045), a manifestation not previously incorporated in the severity score. The severity score was inversely correlated with the fetal hemoglobin (HbF) rate (p = 0.005). Malnutrition (BMI <18.5 kg/m^2^) was present in 47% of patients and was related to the male sex, hip disease (aOR 3.11; p = 0.019) and severe phenotype (aOR 3.53; p = 0.012). Leg ulcers were more frequent in males than in females (p = 0.001; OR 24.3) and were correlated with the number of days of hospitalization (p = 0.029). Hip disease was related to the increasing age (p = 0.008).

**Conclusion:**

In this selected, hospital-based populations of adults with SCA, severe disease was rare, which may be due to survival bias. However, two thirds had moderate severity of the disease, mostly with a low HbF, and they may benefit from HU treatment. In the Central-African setting the separation between vaso-occlusive and hyperhemolytic sub-phenotypes was not applicable.

## Introduction

Sickle cell anemia (SCA) hemoglobinopathy, is the most prevalent monogenic disorder [1]. A recurrent mutation in the beta globin gene (HBB) causes a substitution of glutamic acid at position 6 by valine in the beta-globin chain of hemoglobin [2]. The resulting sickle-hemoglobin (HbS) shows a tendency to polymerize and precipitate in conditions of hypoxia, causing distortion of the red blood cell (RBC) or sickling. This RBC sickling leads to rheological changes, responsible of microvessels vaso-occlusion and painful crises following ischemia. In addition, this distortion causes chronic hemolysis punctuated with severe acute anemia episodes requiring recurrent transfusions. The release of free hemoglobin into the circulation also causes a vasculopathy, including priapism, stroke, maleolar ulcers and chronic kidney disease [3]. In addition to vaso-occlusion and hemolysis, inflammation plays a significant role in the clinical expression of SCA [4]. On the long term, this results in progressive organ damage.

The clinical presentation and the severity of SCA differ from one patient to another and in the same patient over time [5,6]. Some authors describe that patients with SCA may be subdivided according to two sub-phenotypes, vaso-occlusion or chronic hemolysis. The vaso-occlusive phenotype is characterized by a relatively high steady state hemoglobin and frequent vaso-occlusive crises, acute chest syndrome and aseptic necrosis of the femoral head. The hyperhemolytic phenotype on the other hand shows low basal hemoglobin and complications such as stroke, leg ulcers, priapism and renal failure [7].

Environmental factors including socio-economic conditions, nutrition, infection and access to care may influence clinical manifestations of SCA [8]. In high-income countries, infection control and access to comprehensive health care have contributed to the reduction of morbidity and mortality related to SCA. The survival of SCA patients reaches 86% at age 18 in the USA and 99% at age 16 in the UK [6], while mortality in low-income Sub-Saharan countries is 50–90% before the fifth birthday [9]. Other factors could have contributed eg primary care services, universal screening, immunization and patient education, to change the disease expression in Sub-Saharan Africa.

Treatment with hydroxyurea (HU) has been shown to improve acute and chronic manifestations of SCA [10]. This drug is widely used in infants and adults in high-income countries, while its use remains limited in Sub-Saharan Africa despite a high disease burden [11]. Studies on the use of HU in Central Africa are rare and mainly concern children [10–12].

The adult population of SCA patients in Central-Africa is poorly studied and little knowledge exists on HU treatment in adult SCA patients in this region. In view of a potential treatment with HU of adults with SCA in Kinshasa, knowledge on the variability in phenotypic manifestations and insight into factors associated with complications of SCA in adult patients of Central Africa is essential.

To reach this goal, we studied an adult cohort of SCA patients with a genetically confirmed diagnosis. We evaluated the variability of the severity of SCA and the relationship between acute and chronic complications of SCA and laboratory parameters.

## Materials and methods

We conducted a cross-sectional study at the “Centre de Médecine Mixte et d’Anémie SS (CMMASS)” located at the Yolo neighbourhood in Kinshasa, capital of the Democratic Republic of the Congo. This center is dedicated to the management of SCA patients and research in this domain.

The initial study population consisted of adult patients diagnosed with SCA by hemoglobin electrophoresis and regularly followed at CMMASS. Patients were identified from hospital records and were invited by phone to a consultation at CMMASS. Patients were included during a period of 12 months: from September 2017 to end August 2018.

Clinical history was recorded along with data from a complete physical examination to determine whether they were in steady state. Steady state was defined as a state of physiological equilibrium characterized by the absence of an acute painful episode and of an infectious or inflammatory process for at least four consecutive weeks; no history of blood transfusion in the last 4 months; no treatment with medications that may affect the blood counts during the previous 3 weeks [13].

We recorded weight, height, blood pressure, heart rate, respiratory frequency, and oxygen saturation. The nutritional status was evaluated by the body mass index (BMI). The severity of the disease was determined using a severity score based on clinical criteria, as proposed by Mikobi et al. (2015) (Table 1). The score parameters were mainly obtained through the clinical history and physical examination of the patients. Medical imaging (ultrasound, hip radiography and magnetic resonance imaging) were not used routinely because of their high cost. This score distinguishes asymptomatic (ACP, score ≤5), moderate (MoCP, score 6–15) and severe (SCP, score ≥16) clinical phenotypes [14]. In the present cohort, which is hospital-based, patients with a score of 5 or less are symptomatic and thus not totally exempt from acute or chronic complications of SCA and were therefore classified as a mild clinical phenotype (MiCP) instead of ACP.

**Table 1:** Clinical criteria and severity score based on the phenotype (reproduced from Mikobi et al.) [14].

Clinical criteria	Variables	Score (points)	How data was obtained		
			Patient history	Physical examination	Laboratory and medical imaging
Days of hospitalization/year	≤ 1	0	+		
	2–7	2			
	≥8	5			
Severe vaso-occlusive crises/year	0	0	+		
	1–2	2			
	≥3	5			
Blood transfusions/year	0	0	+		
	1–2	2			
	≥3	5			
Hip disease	Absent	0	+	+	[hip x-ray, MRI]
	Present	5			
Leg ulcer	Absent	0		+	
	Present	5		+	
Hepatobiliary complications	Absent	0	+		[Ultrasound]
	Cholecystectomy	2			
	Present	5			
Neurologic events	Absent	0	+	+	[MRI]
	Present	5			
Renal disorders	Absent	0			+
	Present	5			
Body mass index	19–27	0		+	
	< 19	2			

MRI: Magnetic resonance imaging; +: Yes; [] indicate parameters evaluated on clinical indication only.

For each patient, we collected blood in two 4ml EDTA tubes. We obtained a full blood cells count (red blood cells (RBC), white blood cells (WBC) and platelets). Biochemical analyses included lactate dehydrogenase (LDH), bilirubin, serum creatinine, aspartate aminotransferase (AST) and alanine aminotransferase (ALT) (Laboratory of biochemistry and hematology, Faculty of Pharmaceutical Sciences of the University of Kinshasa (UNIKIN)).

Hemoglobin electrophoresis was done at the Laboratory of Human Genetics at UNIKIN using the automate Minicap (Sebia, Phoresis Rel 8.6.3.). DNA was extracted by the salting out method, and mutation analysis for the sickle cell anemia mutation (E6V) was done as previously described [15].

Mutation analysis of the β-globin gene (NG_000007.3) was done by resequencing the coding exons and by MLPA, in a routine laboratory diagnostic setting.

### Ethical approval

The study was approved by the ethical committee of the school of public health of the University of Kinshasa (Approval reference: ESP/CE/079/2016), DRC. A verbal informed consent was obtained from all patients before their inclusion in the study.

The informed consent form written in French and translated into the four national languages (Lingala, Swahili, Tshiluba and Kikongo) was given and explained to the patient in the language of his choice. An appointment was fixed for the return of the form and obtaining the response of the person concerned. Taken the local cultural context into account, a verbal response was sufficient to be included in the study.

### Statistical analyses

Data were recorded in an Excel sheet and analysed with an IBM SPSS Statistics 25.0 software. Categorical data were presented in the form of frequency tables. Quantitative variables were presented by measures of central tendency and dispersion (mean and standard deviation). The Student’s t-test and ANOVA allowed respectively, comparing means in pairs and in different categories. The correlation coefficient was used to measure the degree of relationship between different quantitative variables. Poisson regression was used to test the effect of a few factors on variables of a discrete nature. Logistic regression had made it possible to study the association between a qualitative dependent variable and explicative variables. The level of statistical significance was defined by a p value less than 0.05.

## Results

**Fig 1:**
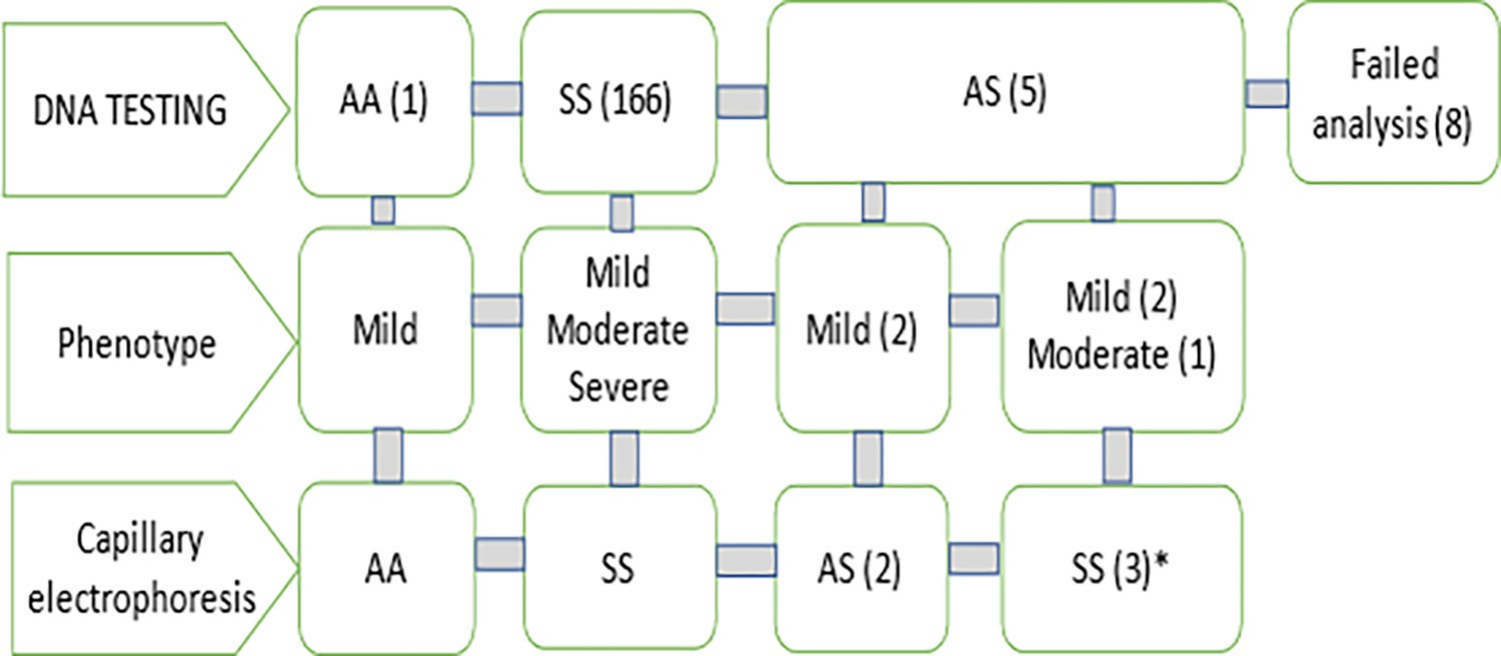
Diagnostic evaluation in 180 patients followed for sickle cell disease.

We recruited 180 patients followed for SCA in CMMAS. The SCA status was evaluated using a DNA test and Hb capillary electrophoresis (Fig 1). The diagnosis of homozygous SS status was confirmed in 166 patients. One patient has a normal hemoglobin AA, which was confirmed by Hb electrophoresis. This patient had a clinical score of 2 which corresponds to a mild phenotype. Five patients were heterozygous AS by DNA analysis, of whom three were confirmed SCA after Hb electrophoresis. The latter three had a HbF > 5%, with a HbA_2_ >3,4%, an absence of HbA_1_ and a Hb 9-12g/dl in steady state. They have a variant of sickle cell disease, i.e. a compound heterozygosity for an S allele and a β thalassemia mutation (HbS/Beta° thalassemia). In two patients, whose clinical phenotype was moderate for one and mild for the other, we identified, the c.315+1G>A variant in intron 2 of the HBB gene (rs33945777). The third patient had a mild clinical phenotype and carried c.92+2T>C variant in intron 1 (rs33956879). Both variants are classified as pathogenic for Beta thalassemia in Clinvar (two stars). The analysis failed in 8 due to clotting of the blood sample.

The diagnosis of SCA was evaluated by a combination of DNA analysis and Hb electrophoresis. This revealed 1 case without SCA, two heterozygous patients and three with HbS/Beta° thalassemia (*).

The study group thus consisted of 166 genetically confirmed SCA patients.

Median age was 24 years (range 18–40 years). There were 107 (64.5%) females and 59 (35.5%) males (sex ratio M/F: 0.55).

**Fig 2:**
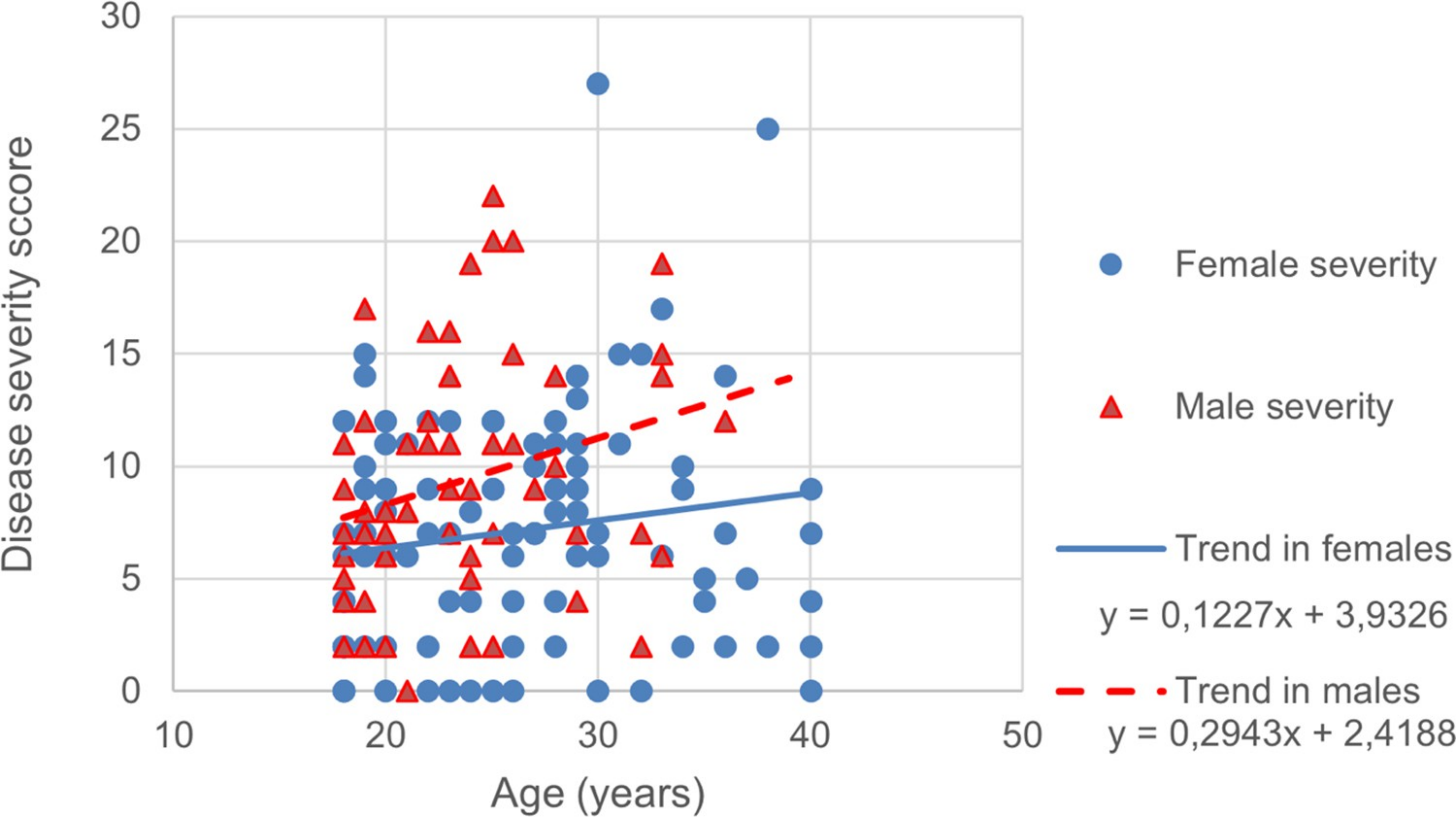
Distribution of disease severity score according to age and sex.

**Fig 3:**
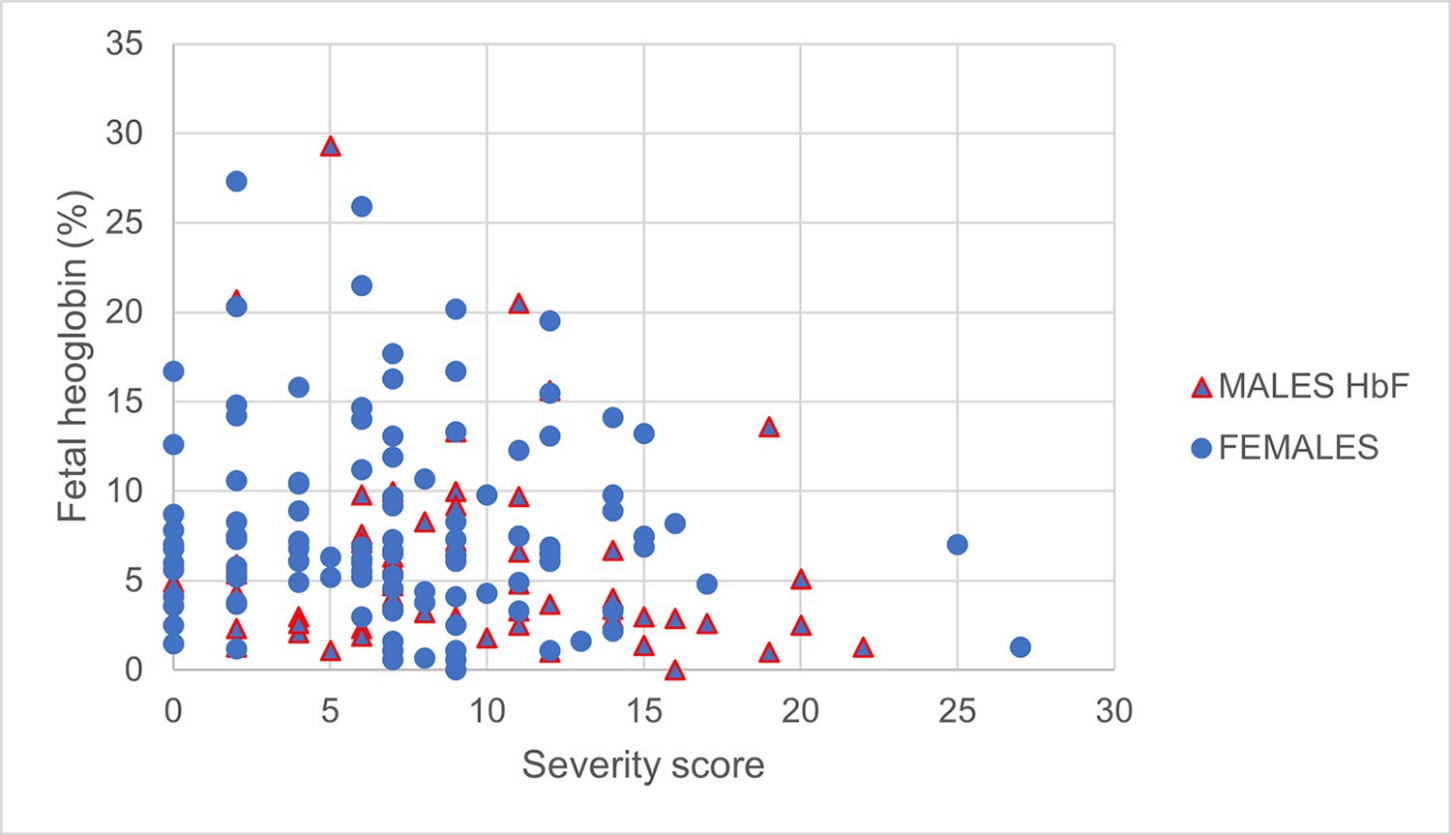
Fetal hemoglobin and severity score according to sex.

The disease severity defined by a clinical score was mild in 48 (28.9%), moderate in 107 (64.5%) and severe in 11 (6.6%). The disease severity score increased with patient’s age (p ≤ 0.001), with median age of 24 years (range 18–40 years) in the MiCP and MoCP, and 25 years (range 19–38 years) in the SCP group (Fig 2). The severity was higher in males compared to females (average score F: 7.2±5.1, M 9.3±5.2; p = 0.012) with a sex-ratio of 0.4 in the MiCP group, 0.6 in the MoCP and 2.7 in the SCP (Fig 2). Almost all male patients (56/59) had a HbF rate less than 10% (Fig 3).

The severity score was inversely correlated with the HbF rate (p = 0.015) (Table 2, Fig 3). Other hematological parameters or markers of hemolysis (LDH, Bilirubin) did not show a correlation with the severity score (Table 2).

**Table 2:** Biological parameters associated with severity score of SCA.

Parameters	p	IRR (CI 95%)
HbF (%)	**0.015**	0.98 (0.97–1)
Hematocrit (%)	0.011	1 (1–1.02)
Reticulocytes (/mm^3^)	0.775	1 (1–1)
WBC (/mm^3^)	0.397	1 (1–1)
Neutrophils (%)	0.600	1 (0.99–1)
Platelets (/mm^3^)	0.387	1 (1–1)
LDH (IU/L)	0.966	1 (1–1)
Total bilirubin (mg/dl)	0.303	0.98 (0.96–1)

HbF, fetal hemoglobin; IRR, incidence risk ratio; WBC, white blood cells; LDH, lactate dehydrogenase.

Mean BMI was 18.6±2.2kg/m^2^ and was not significantly different according to severity with 18.5±1.8 in MiCP, 18.7±2.4 in MoCP and 18.6±2.1 in SCP. Malnutrition (BMI <18.5 kg/m^2^) was present in 47% of patients. In a multivariate analysis, malnutrition was associated with male sex (aOR 3; p = 0.006), hospitalization 2 or more days/year (aOR 3.5; p = 0.024), hip disease (aOR 3.7; p = 0.027) and severe phenotype aOR (7.21; p = 0.001) (Table 3).

**Table 3:** Factors associated with malnutrition (BMI<18.5 kg/m^2^).

Variables	Multivariate analysis	
	p	aOR (95%CI)
Age groups (years)		
≤ 25		1
> 25	0.178	1.7(0.79–3.54).
Sex		
Female		1
Male	**0.006**	3,1(1.38–6.73)
Hospitalization (Days/years)		
2–7	**0.024**	3.5 (1.18–10.78)
≥8	**0.002**	7.3 (2.08–25.63)
Vaso-occlusive crises	0.16	0.93(0.83–1.03)
Transfusion	0.222	0.9(0.77–1.06)
Hip disease		
Present	0.027	3.7(1.16–11.89)
Leg ulcers		
Present	0.425	1.76(0.44–7.04)
Severity		
moderate	**0.004**	6.36(2.7–9.49)
severe	**0.001**	7.21(3.3–13.7)
Hemoglobin	0.755	1.03(0.86–1.23)

Biological data (Table 4) showed normocytic normochromic and regenerative anemia with low fetal hemoglobin rate (7.3±5.5%). The HbF level did not vary with age (p = 0.845; r 0.014).

**Table 4:** Summary of the biological parameters.

Parameters	Normal values	Mean values ± SD		p-value
		Females	Males	
Hb (g/dl)	12–18	7.9±1.7	8.5±2.6	0.082
MCV (fl)	75–96	83.8±10.1	81.6±9.7	0.194
MCH (pg)	27–32	27.9±3.7	27.3±4.1	0.365
WBC (x10^3^/mm^3^)	40–10	12.5±9.7	12.8±10.1	0.844
Neutrophils (%)	50–70	40.4±14.4	40.2±16.8	0.658
Platelets (10^3^/mm^3^)	150–450	461±206	496±231	0.364
Retic (10^3^/mm^3^)	20–80	139±90	145±96	0.704
HbF (%)	1–2	8±5.5	5.9±5.3	**0.019**
Bili T (mg/dl)	0–1.1	2.6±2	3.6±2.9	**0.011**
Bili D (mg/dl)	0–0.3	0.6±0.7	0.7±1	0.319
LDH (IU/L)	230–460	1555±895	1627±593	0.582

Hb, hemoglobin; MCV, mean cell volume; MCH, mean corpuscular hemoglobin content; WBC, white blood cells; Retic, reticulocytes; HbF, fetal hemoglobin; Bili, bilirubune; LDH, lactate dehydrogenase.

Chronic complications were observed in a total of 56 patients (33.7%), including hip disease in 25 patients (15%), leg ulcer in 18 patients (10.8%), hepatobiliary complications in 10 patients (6%) and a neurological event in 4 patients (2.4%). Eight male patients (13.6%) had priapism. In males, the severity score was correlated with the presence of priapism (p = 0.045).

Nine patients presented more than one chronic complication. Five of the patients with hip disease presented associated complications, i.e. leg ulcers in 3 (1.8%), or hepatobiliary complications in 2 (1.2%).

The risk of any complication was positively associated with increasing age (p = 0.007) and being male (p ≤ 0.001). Taken individually, leg ulcers were more frequent in males than in females (p = 0.001; OR 8) (Table 5).

**Table 5:** Factors associated with leg ulcer.

Model 1: Clinical parameters			Model 2: Biological parameters		
Parameters	p	aOR (CI)	Parameters	p	aOR (CI)
Male sex	0.001	8.1(2.45–26.21)	Hematocrit (%)	0.23	1.06(0.96–1.17)
Age (years)	0.516	1.03(0.94–1.14)	MCV (fl)	0.045	0.95(0.89–0.99)
Hospitalization	0.097	1.02(0.99–1.04)	Retic (/mm^3^)	0.39	1 (0.93–1.04)
Priapism	0.258	0.22(0.02–2.99)	Platelets (/mm^3^)	0.96	1(0.96–1.2)
VOC	0.589	1.04(0.90–1.20)	WBC (/mm^3^)	0.885	1(0.94–1.06)
Transfusions	0.422	0.89(0.69–1.17)	Neutro (%)	0.1	0.97(0.93–1.01)
			LDH (IU/L)	0.743	1(0.93–1.01)

VOC, vaso-occlusion crises; MCV, mean cell volume; Retic, reticulocytes; WBC, white blood cells; Neutro, neutrophils; LDH, lactate dehydrogenase.

The Table 5 shows that, the hip disease was more frequently observed with increasing age (p = 0.023) (Table 6).

**Table 6:** Factors associated with hip disease.

Model 1: clinical parameters			Model 2: biological parameters		
Parameters	p	aOR (CI)	Parameters	p	aOR (CI)
Male sex	0.123	2.1(0.82–5.38)	Hematocrit (%)	0.186	0.94(0.85–1.03)
Age (years)	0.023	1.1(1.01–1.18)	MCV (fl)	0.662	0.99(0.95–1.04)
Hospitalization	0.168	0.96(0.90–1.02)	Retic (/mm^3^)	0.328	1 (0.92–1.06)
VOC	0.775	1.02(0.90–1.15)	Platelets (/mm^3^)	0.822	1(0.98–1.06)
Transfusions	0.202	1.13(0.94–1.36)	WBC (/mm^3^)	0.989	1(0.99–1.03)
			Neutro (%)	0.133	1(0.99–1.06)
			LDH (IU/L)	0.875	1(0.99–1.07)

VOC, vaso-occlusion crises; MCV, mean cell volume; Retic, reticulocytes; WBC, white blood cells; Neutro, neutrophils; LDH, lactate dehydrogenase.

## Discussion

The clinical expression of SCA varies widely from one individual to another [5,6]. Some patients have an almost normal life, while others have a very poor quality of life due to the frequency of hospitalizations and the occurrence of chronic complications. In this study, we evaluated factors associated with the severity in a cohort of young adult patients.

First, our data stress the need to confirm the diagnosis of SCA by means of a DNA test; indeed, a small number of patients had no SCA, but rather sickle cell disease (SCD) variants.

Our cohort consisted of young adult patients, with a median age of 24 years (range 18–40 years). This age is relatively high if we consider that up to a few decades ago, the life expectancy of sickle cell patients was very low in Africa, where 50–90% would die due to a lack of access to proper treatment [9]. The median age of our patients is close to the median survival age of 33 years observed in a Tanzanian hospital cohort [16]. This reflects an improvement in the quality of care currently provided to SCA patients [6]. The patients included in this study were recruited in a hospital dedicated in the care for persons with SCA. Therefore, the severity is likely to be biased towards patients with more pronounced manifestations, with two thirds presenting a moderate phenotype. On the other hand, patients with a severe phenotype are underrepresented (only 6.6%), most likely due to the high mortality among this group of patients in low-resource countries where access to health facilities is difficult.

Patients with a mild phenotype have less need of hospital care compared to the more severe phenotypes. In the present study, they belong to a minority of Congolese SCA patients who have access to a regular follow-up at the hospital and could thus be recruited for this study.

We have applied the clinical severity score that was recently proposed by Mikobi et al [14]. This score is based on clinical manifestations and reflects the impact of the disease on the person’s health. Not unexpected was the positive association between age and disease severity (p≤0.001). With increasing age, patients develop chronic complications that increase their severity score. Male patients were more severely affected than females. This difference observed in the severity of clinical expression of sickle cell anemia is thought to be due to the bioavailability and the response to nitric oxide (NO) which are larger in women than in men. NO has a vasodilator effect, it inhibits the production of vascular cell adhesion molecule 1 (VCAM1) by endothelial cells and limits platelet aggregation. In women of childbearing age, the production of NO is stimulated by estrogen, explaining their being relatively more protected against vaso-occlusive phenomena and resulting harmful effects on the organs [17]. In the present cohort, males are underrepresented, especially in the mild and moderate group. This may again be due to recruitment bias, with males presenting for follow-up or treatment only with more severe manifestations. In a system where there is no national health insurance system, as in the DRC, costs are usually shared by family members. Adult females may be more supported by this family network than males. On the other hand, a higher morbidity and mortality has also been described in males with SCA in the USA [18].

Priapism is not included in the proposed severity score, since it is limited to males. However, the presence of priapism was associated with an increase of the disease severity, which indicates that this could also be included as a clinical parameter for severity of SCA in males. The literature reported that priapism is associated with fivefold risk of onset of pulmonary hypertension in male patients [7].

Elevated levels of HbF have a protective effect, primarily by inhibiting the sickling of RBC. This is correlated with a reduction in morbidity and mortality [18–20]. Also in our cohort, the severity of SCA was negatively associated with the HbF levels (p = 0.005). Mean HbF was 7.3±5.5% and in accordance with previous studies conducted in the DRC among pediatric patients followed in a comprehensive program (HbF 7.2±5%) and Congo-Brazzaville (HbF 8.8±5.8%) [21,22]. A level of HbF of 10% or more is considered to be protective against chronic complications, and a level of HbF of 20% or more was proposed as protective against recurrent complications such as infections and acute chest syndrome [23]. The low HbF levels observed in this cohort can be explained by the fact that we studied a clinically affected population. However, another contributing factor may be the likely predominance of the haplotype Bantou in this geographical region, which is characterized by a low rate of HbF (<10%) and a severe disease [21,22]. As previously reported, we also observed significantly higher HbF levels in females than in male [21]. This could partly explain the marked severity in male than in female (p = 0.012). The difference could be attributed to a gene linked to Xp22.2 [24]. However, the clinical expression of the SCA is not only related to the level of HbF. The pathophysiology is complex and involves several factors, including environmental factors such as colder climate, infections [8] or the co-inheritance of another hemoglobinopathy (alpha thalassemia) [20].

SCA is a chronic disease, with a high mortality and increasing morbidity with age. Malnutrition, defined as BMI <18.5 kg/m^2^ was more frequent in patients hospitalized for least 2 days per year, patients with hip disease, severe phenotype and the male sex. A more severe phenotype is associated with frequent hospitalizations that could impact the nutritional status of patients because episodes of hospitalization are accompanied by anorexia related to pain or infection, resulting in a reduction in food intake. The data in this cohort are in accordance with other studies, which reveal that male sex was associated with an increased risk of malnutrition [25,26]. However, the cause of this difference between males and females is not clearly established. A previous study in the DRC of healthy individuals aged 18 years or older also revealed a higher prevalence of malnutrition in males (18.8%) than females (13.1%) [27].

Chronic complications were found in one third of patients. Leg ulcers were seen in 11% of cases. A multicentric study of leg ulcer in SCA patients reported a prevalence of 2.4% in USA, 3.5% in Italy and 18.6% in Ghana [28]. Among these Ghanaian patients, 14.9% were adults. As reported, leg ulcer is more frequent in tropical regions, is associated with the male sex and its prevalence increases with patient age [29]. In the present study, we did not observe an age effect on the prevalence of leg ulcer, but this may be due to the small number of affected patients.

Hip disease is very common in adult SCA with frequencies exceeding 30% [30,31]. The frequency of 15% observed in our study is certainly an underestimate because systematic imaging of the hips is not available to most SCA patients in follow-up. Thus, the subclinical stages of this pathology are not recognized, and cases are only diagnosed at an advanced, symptomatic stage. Hip disease has been reported to be due to increased blood viscosity, sickling of RBC and its consequences (21). However, we found no significant association with either the frequency of vaso-occlusive crises or the hematocrit level as described before [7].

Previously, it has been proposed that SCA patients could be categorized depending on their predominant phenotype, characterized by either vaso-occlusion or hyperhemolysis [7,32]. In our cohort, patients with leg ulcers, supposed to show an hyperhemolytic phenotype did not have significant association with other features constituting this sub-group: frequent transfusions, priapism, and elevated LDH levels. Similarly, hip disease, mainly due to vaso-occlusion, did not show a consistent association with hematocrit and the number of vaso-occlusive crises. In our study, we observed that vaso-occlusion and hemolysis may coexist in the same patient and their balance may vary over time. Larger studies are needed to explore the question whether separation in vaso-occlusive or hyperhemolytic sub-phenotypes is appropriate in the Central-African environment.

## Conclusion

The assessment of the severity of SCA is of great interest, mainly in a low-income country. The physician needs to evaluate the impact of the disease on the patient’s health in order to give the most appropriate treatment and follow-up. Hydroxyurea treatment can be initiated to increase the production of HbF and thus reduce the severity of SCA. In addition, the severity score will also allow the evaluation of the response to HU treatment.

## Supporting information

S1 Data (XLSX)
